# Millimeter-precision positioning for wide-angle indoor area enabled by metalens-integrated camera

**DOI:** 10.1515/nanoph-2024-0277

**Published:** 2024-08-29

**Authors:** Muyang Li, Yue Wu, Haobai Li, Zi-Wen Zhou, Yanxiang Zhang, Zhongyi Yuan, Zaichen Zhang, Ji Chen

**Affiliations:** National Mobile Communications Research Laboratory, School of Information Science and Engineering, Frontiers Science Center for Mobile Information Communication and Security, 12579 Southeast University, Nanjing 210096, China; Purple Mountain Laboratories, Nanjing 211111, China

**Keywords:** high-precision positioning, wide-angle imaging, integrated meta-device, imaging processing

## Abstract

Due to signal shielding caused by building structures, conventional mature positioning technologies such as the Global Positioning System (GPS) are only suitable for outdoor navigation and detection. However, there are many scenarios that urgently require high-precision indoor positioning technologies, such as indoor wireless optical communications (OWCs), navigation in large buildings, and warehouse management. Here, we proposed a millimeter-precision indoor positioning technology based on metalens-integrated camera, which determines the position of the device through imaging of beacon LEDs. Thanks to the wide-angle imaging design of our metalens, the camera can accurately capture images of beacon LEDs even when it is situated in distant corner locations. Consequently, our localization scheme achieves millimeter-level positioning accuracy across majority of wide-angle (∼120°) indoor area. Compared to traditional positioning schemes by photodiode (PD), our imaging-based approach demonstrates superior resistance to interference, thereby safeguarding positioning precision from the external signals influence. Furthermore, the compact dimensions and high performances of the positioning device make it suitable for integration into highly portable devices, such as smartphones and drones, revealing its broad potential applications in the future.

## 1 Introduction

Location information is of great importance in many applications or services, for it determines the accuracy and efficiency of information transmission [1], [2], [3]. The widely used global positioning systems (GPS) face challenges in indoor or obstructed environments, due to factors such as electromagnetic shielding, multipath fading, and obstacles blocking signals [4], [5]. To address the indoor operational blind spots of GPS, various wireless positioning solutions have been developed, such as wireless local area networks (WLAN), radio frequency identification (RFID), ultra-wideband (UWB), etc. [6], [7], [8], [9], [10]. However, the development of these solutions is limited by the electromagnetic interference, noise, stability, cost, and other factors [11]. With the significant advancement of light-emitting diodes (LEDs) as light sources, visible light communication (VLC) technology based on LEDs has been extensively researched in recent years [12], [13], [14], [15]. The positioning technologies based on VLC is a kind of green service, and attracts much attentions, which possesses advantages of electromagnetic interference resistance, system stability, and relative high positioning accuracy [16], [17], [18], [19]. In addition, the positioning devices can also integrate with lighting systems, significantly reducing construction and operational costs.

VLC positioning technologies can be broadly categorized into two types, one is the geometric analyze methods based on intensity measurement and the other is the imaging methods by cameras [20], [21]. Geometric methods are generally based on analyzing the characteristics of VLC signals received through photodiodes (PD), which establish geometric relationships for positioning measurement using parameters such as time of arrival (ToA), angle of arrival (AoA), angle difference of arrival (ADoA), and received signal strength (RSS). However, some of these methods require strict synchronization clock cycles, leading to higher equipment costs. Additionally, the measurement accuracy of this type is susceptible to interference from noise generated by other external light sources [22], [23], [24], [25], [26]. In comparison, the imaging methods analyze the location information from the lens imaging relationship between objects and images. The large number of pixels on imaging sensors guarantees the higher positioning accuracy than geometric analyze methods. However, to achieve high-quality imaging, complex lens assemblies are needed, resulting in a larger overall volume of the positioning device, which is less conducive to integration into mobile devices. Furthermore, with an increase in the detection angle, not only does the detection intensity decrease significantly but the original positioning analysis models also become ineffective, which severely restricts the detection range of both two types of positioning technologies [27], [28].

In this work, we proposed a three-dimensional (3D) positioning scheme based on a metalens-integrated camera, which takes full advantages of the ultra-light and ultra-thin properties of metasurface. Metasurfaces are capable of effectively manipulating multidimensional properties of light on an ultra-thin surface, including amplitude [29], phase [30], [31], polarization [32], and orbital angular momentum [33]. Various compact optical functional devices have been realized based on metasurfaces, such as metasurface-based optical detectors, metalens-integrated cameras, or microscopy [34], [35], [36], [37], [38], [39], [40], [41], [42], etc. Distance measurement has also been realized by using metasurface-based devices [43], [44]. However, these devices only achieved one-dimensional positioning function. Three-dimensional positioning based on metasurfaces has not yet been proposed. The metalens-integrated positioning device (MPD) we designed is highly compact in size, facilitating easy integration into mobile devices. The scheme also utilizes the flexible phase design advantage of metasurfaces, realizing a planar wide-angle imaging metalens that can accurately images LEDs over a large angular range (∼120°). Additionally, to address the issue of reduced image intensity of LEDs at large viewing angles, we introduced an analysis algorithm for precisely determining the imaging positions of LEDs, which further reduces the impact of noise on positioning accuracy. The positioning device we proposed combines the advantages of compact size, high positioning accuracy, large detection range, and low cost, providing a revolutionary solution for future high-precision indoor positioning devices.

## 2 Results and discussion

### 2.1 Working principle and architecture of the MPD

**Figure 1:**
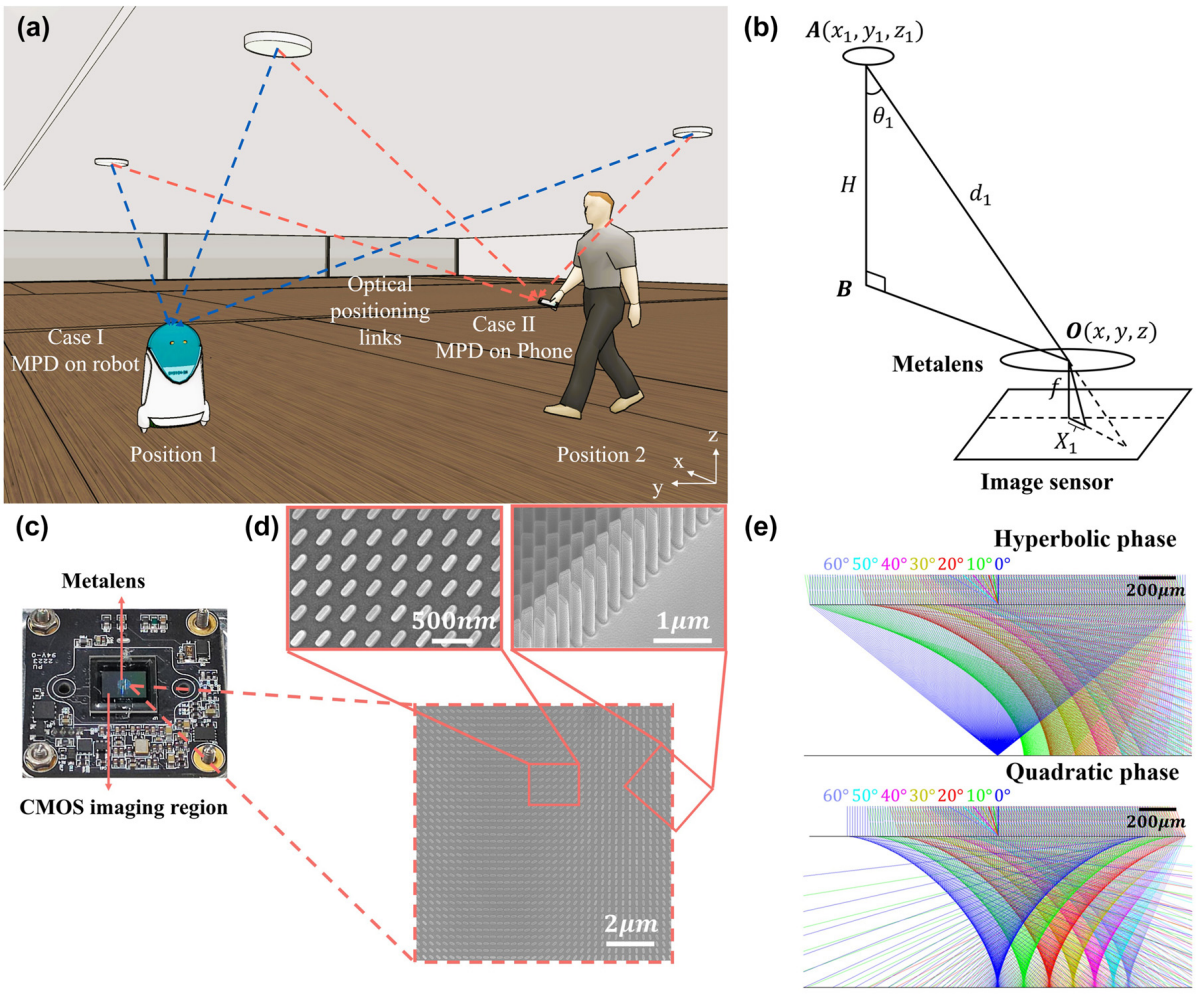
The positioning device and its working principle. (a) Schematic of the MPD application scenario. The MPD can be integrated into the mobile phone or robot and then determine its own position by imaging the beacon LEDs with known locations on the ceiling. (b) Working principle of the MPD. The position of the device is related with the imaging positions of beacon LEDs on the CMOS sensor. *f*: metalens focal length, *X*_1_: distance between the image of the *i*-th LED and the center point of CMOS sensor, *d*_1_: distance between the *i*-th LED and the center point of metalens. (c) The photograph of the MPD, in which the CMOS imaging region and the metalens region are marked. (d) The SEM images of the fabricated metalens structures. (e) The simulated focusing light rays of hyperbolic phase metalens and quadratic phase metalens. The different colors from left to right represent the light rays with incident angles of 0°, 10°, 20°, 30°, 40°, 50°, and 60°, respectively.

The application scenario of the MPD is shown in Figure 1(a). The MPD can be integrated into the mobile phones or moving vehicles and images the LED lights deployed on the ceiling with known positions. Through the geometric relationship of metalens imaging process, as shown in Figure 1(b), the specific position of the device can be deduced by the following equation,

$$\tan^{2}\theta_{i}=\frac{{\left(x-x_{i}\right)}^{2}+{\left(y-y_{i}\right)}^{2}}{{\left(z-z_{i}\right)}^{2}},$$

(1) $$X_{i}=F\left(\theta_{i}\right),$$

where (*x*, *y*, *z*) is the coordinates of the mobile device that is needed to be calculated, (*x*_*i*_, *y*_*i*_, *z*_*i*_) is the coordinates of the *i*-th LED, and *θ*_*i*_ is the included angle between the normal line and the line connecting point (*x*_*i*_, *y*_*i*_, *z*_*i*_) and point (*x*, *y*, *z*). ***X***_***i***_ is the image position of the *i*-th LED relative to metalens center on CMOS sensor, which is related to the angle *θ*_*i*_. Equation (1) indicates that if the imaging positions ***X***_***i***_ were designed in advance, the unknown (*x*, *y*, *z*) would be derived from geometric relationships of three LEDs and their corresponding images.

In traditional geometric optics imaging model, it is assumed that the direction of light remains unchanged when it passes through a lens center. Therefore, the relationship between the imaging position and the incident angle conforms to the condition ***X***_***i***_ = *f* tan*θ*_*i*_. However, this condition is only satisfied when the incident angle *θ* is small, because when *θ* comes larger, non-negligible imaging aberrations (mainly coma) will appear [38], resulting in a significant imaging position deviation from theoretical *f* tan*θ*. Therefore, using a traditional lens for positioning will lead to significant positioning errors beyond a certain viewing angle range. Although the utilization of commercial fisheye lenses can enhance the imaging viewing angle, the bulky and heavy properties present challenges in terms of integration into mobile devices, like smartphones.

Benefitting from the ultra-thin, ultra-light, and flexible phase design advantages, metalens with only a single-layer of structure can realize the function comparable to conventional bulk refractive lenses, providing a promising way for miniaturization of positioning devices. In this work, we design a quadratic phase metalens that meets the imaging requirements of wide-angle and aberration-free, the phase profile of which is expressed as

(2) $$\phi \left(x,y\right)=-\frac{k}{2f}\left(x^{2}+y^{2}\right),$$

where *k* is the wave number, and *f* is the metalens focal length. When an extra phase caused by oblique incidence is imposed, the quadratic phase profile would translate the linear extra phase into a spatial shift, shown as

(3) $$\begin{array}{cc} \phi \left(x,y\right) & =-\frac{k}{2f}\left(x^{2}+y^{2}\right)-kx\sin\theta \\ & =-\frac{k}{2f}\left[{\left(x-f\sin\theta \right)}^{2}+y^{2}\right]+\frac{fk\sin^{2}\theta }{2}\cdot \end{array}$$

As a result, the effect of oblique incidence is a spatial translation of the focal spot equals to *f* sin*θ*, as shown in Figure 1(e). In this work, the metalens is designed with a diameter of 1 mm, a focal length of 450 μm, and working wavelength of 470 nm, making the theoretical viewing angle reaches ±90°.

The metalens was fabricated in silicon nitride (Si_3_N_4_) nano-posts on a SiO_2_ substrate using standard electron-beam lithography (EBL) and dry etching (see Methods for details). The meta-atoms are geometric phase type designed in wavelength of 470 nm, which are 240 nm in length, 80 nm in width, 800 nm in height, and 300 nm in period. The geometric phase metasurface can be used to filter the environment noise, when the noise significantly interferes the imaging of beacon LEDs (see Supplementary Note S1 for detailed discussion). The modulation phase of the meta-atom is related with the rotating angle. Figure 1(c) shows the physical photograph of the MPD, in which the metalens is directly mounted upside down on the CMOS sensor (Imaging source, DMM 27UJ003-ML, pixel size: 1.67 μm) and fixed by an optically clear adhesive (OCA) tape (Tesa, 69402). The thickness of the OCA tape was chosen equals to the metalens focal length, so as to ensure the far-field objects could be clearly imaged on the CMOS sensor. The size of the entire device is 3 cm × 3 cm × 0.3 cm, while the main photosensitive area measures 1 cm × 1 cm, demonstrating significant advantages in volume compared to traditional imaging devices. Figure 1(d) shows the scanning electron microscope (SEM) image of part of the wide-angle metalens, the two zoom-in pictures are the top view and side view of structure details, respectively. The SEM images illustrate that the processed structure retains optimal design dimensions and impressive steepness.

Figure 1(e) shows the Zemax OpticStudio simulated focusing performances of hyperbolic phase metalens and quadratic phase metalens, respectively, at different incident angles. The different colors from right to left sequentially represent the light rays with incident angles of 0°, 10°, 20°, 30°, 40°, 50°, and 60°, respectively. The simulation results reveal that the hyperbolic phase metalens can perfectly focus the normally incident light. However, as the incident angle increases, the focusing performance deteriorates rapidly. On the contrary, for the quadratic phase metalens, although some stray light may be present during the focusing of light at each incident angle, the majority of the energy from even large-angle incident light can still be focused. This is why our device is capable of achieving good-quality imaging of wide-angle LED lights and exhibits high positioning accuracy.

### 2.2 Characterization of the MPD imaging performance

**Figure 2:**
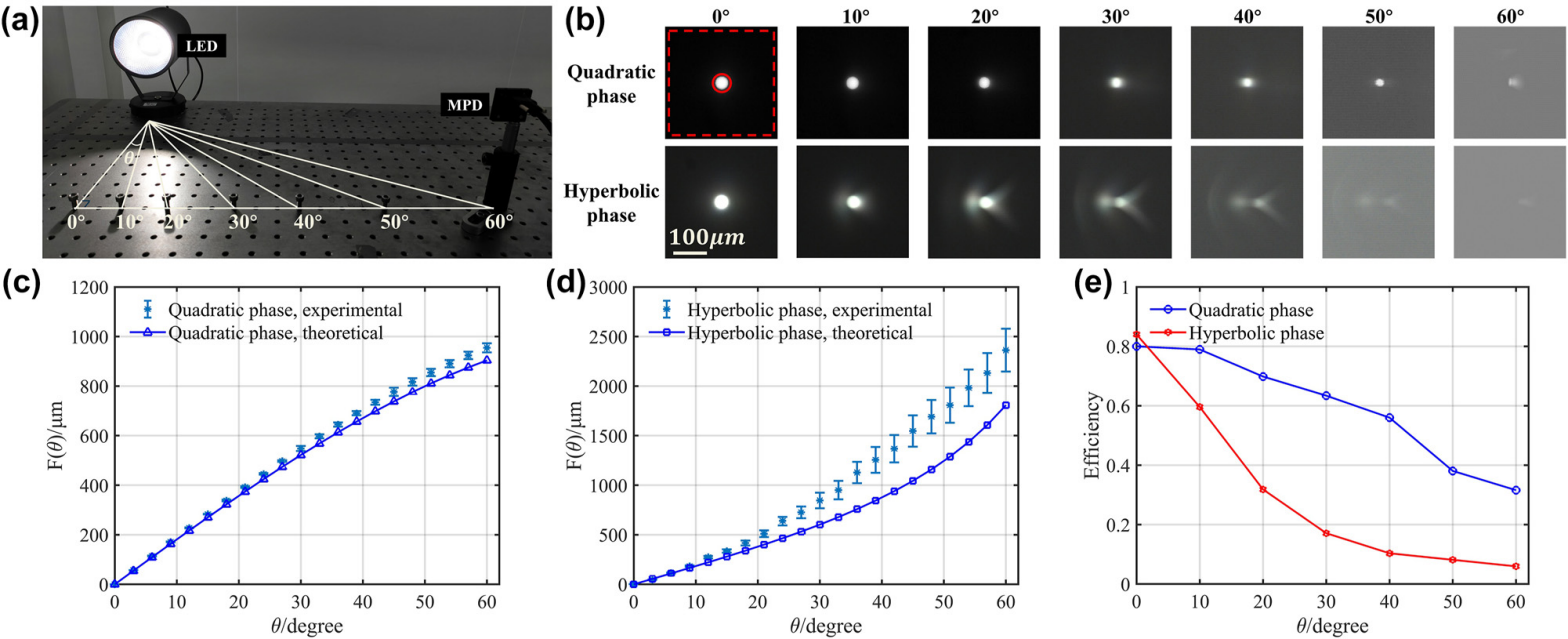
Imaging performance comparison of the MPD and the hyperbolic phase metalens at different viewing angles. (a) Experimental setup for imaging of the LED while the imaging device is placed at different viewing angles. The viewing angles are marked below the position. (b) Quadratic phase and hyperbolic phase metalens imaging results corresponding to different incident viewing angles from 0° to 60°. The red circle and red square represent the focusing region and observed region, respectively. The measured and theoretical imaging positions of (c) quadratic phase metalens (the MPD) and (d) hyperbolic phase metalens at different viewing angles. The abscissa represents the LED viewing angles, while the ordinate (blue marked line & error bar) represents the distance from the imaging point to the CMOS center. (e) Focusing efficiency of quadratic phase and hyperbolic phase metalens at different viewing angles.

To demonstrate the positioning performances of the MPD, we first characterized its imaging of a fixed beacon LED when it was placed at different viewing angles. The experimental setup is shown in Figure 2(a). The LED was fixed at a specific position and height on the optical platform, while the MPD was placed at a line with the same longitudinal distance and height of the LED. The relative viewing angle between the MPD and the LED was altered by adjusting the lateral distance. For comparison, a metalens with hyperbolic phase profile, which possesses diffraction limited imaging capability, was also implemented to image the LED at different viewing angles. Figure 2(b) shows the comparison of LED images captured by the MPD and the hyperbolic phase metalens at viewing angles of 0°, 10°, 20°, 30°, 40°, 50°, and 60°, respectively. It is readily apparent that the shapes and intensity of MPD images are well preserved, whereas hyperbolic metalens images exhibit noticeable coma aberrations. Moreover, as the viewing angle increases, the intensity of the hyperbolic phase metalens decays rapidly. We demonstrate the intensity decrease quantitatively by using focusing efficiency, which is defined as the ratio of energy within the focusing region (solid red circle in Figure 2(b)) to the energy within the observed region (dashed red square in Figure 2(b)). The focusing efficiencies shown in Figure 2(e) indicate that the quadratic phase metalens has much better focusing property, especially for large-angle incident light. All these factors contribute to the difficulty in determining the positions of imaging spot in hyperbolic phase metalens, resulting in a significant localization error. Figure 2(c) and (d) show the comparison of the experimental detected image positions and the theoretical model at different viewing angles both for the MPD and the hyperbolic metalens. For the MPD, the experimental results align well with the theoretical model at various angles, while for hyperbolic metalens, when the viewing angle gets larger, the experimental results will extremely deviate from the theoretical model. This is why our MPD can achieve high-precision positioning performance across wide viewing angle ranges, while traditional lenses perform poorly in positioning within such extensive viewing angles.

### 2.3 Indoor positioning detection system

**Figure 3:**
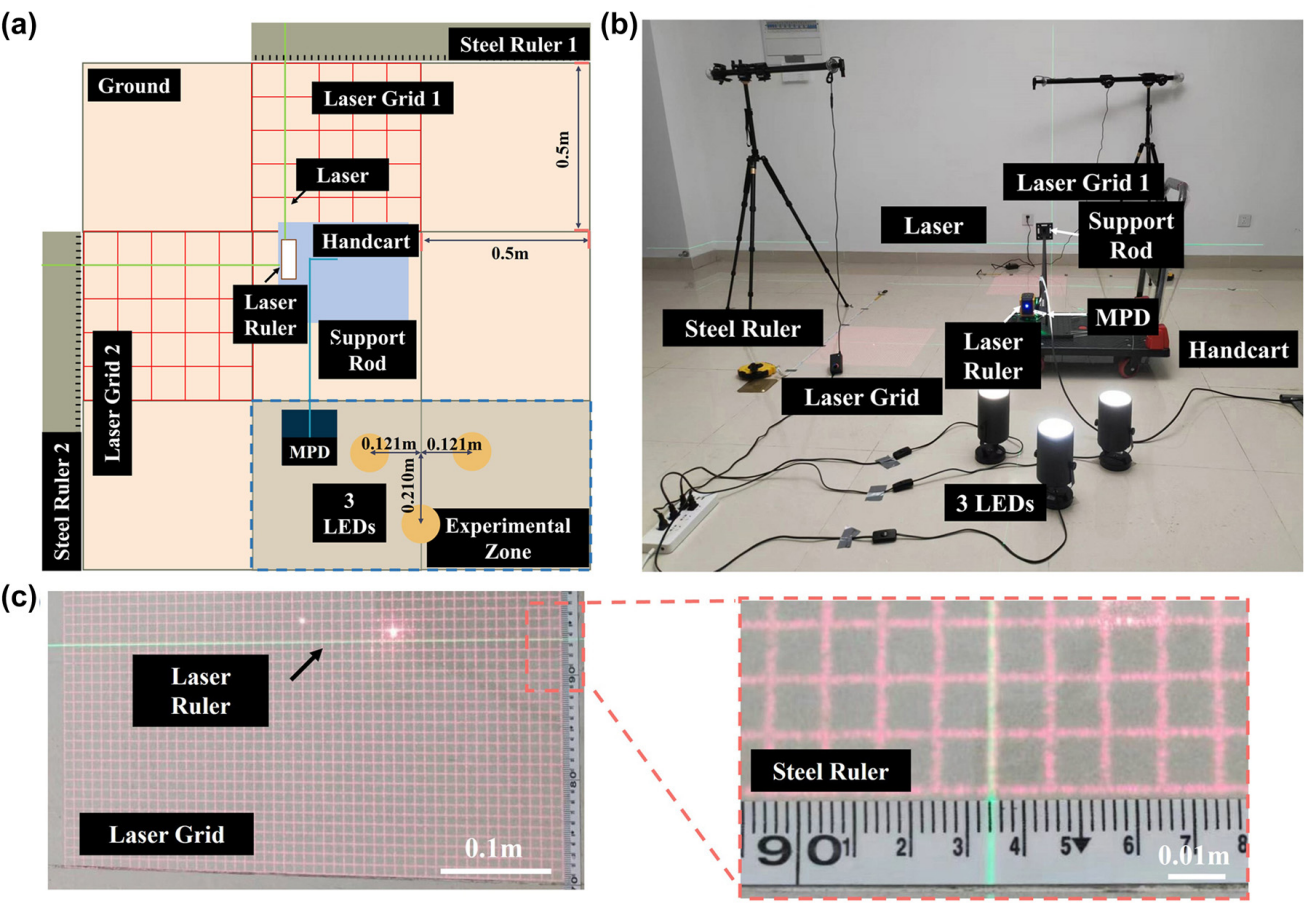
Indoor positioning detection system. (a) Schematic diagram of the positioning solution and the related setups. (b) The physical diagram of the experimental setups and their indoor layout. (c) Schematic diagram for reference position reading, reducing reading errors by aligning the laser ruler with the laser grid.

To experimentally assess the accuracy of our positioning scheme, it is essential to compare its test results with ground truth coordinates. However, determining ground truth coordinates within indoor spaces is not straightforward, as it requires expensive instruments such as ultra-high-definition motion capture cameras or sophisticated laser rangefinders. In the absence of such complex equipment, measurements using a ruler are necessary to precalibrate the coordinates of each position. However, this method not only consumes a significant amount of time and effort but also introduces considerable errors. Here, we propose a cost-effective and easily implementable solution capable of determining high-precision ground truth coordinates. The schematic of our solution is depicted in Figure 3(a), where each large square represents a tile area of 0.5 m × 0.5 m. The experimental testing area is outlined by the blue dashed rectangle in the lower right corner, covering an area of 1 m × 0.5 m, corresponding to imaging viewing angles exceeding ±60°. Three LEDs are arranged in an equilateral triangle configuration at the center of the experimental testing zone, with the distances between each LED illustrated in the figure. The MPD is affixed to a movable handcart (indicated by the blue rectangle) with a long support rod, enabling the MPD to move within the experimental testing zone while the handcart remains outside the zone.

A laser ruler is also affixed to the handcart, emitting two perpendicular green laser beams directed toward steel rulers placed at the room edges, facilitating determination of the MPD and handcart positions in the *XY* plane. To prevent testing errors due to handcart rotation, it is crucial to ensure the green laser beams project vertically onto the steel rulers. Two sets of red laser grids with 50 × 50 square units are projected, aligned parallel to the steel rulers. During testing at each coordinate position, we verify the alignment of the green laser beams with the red laser grids to ensure vertical projection onto the steel rulers, enabling accurate MPD position readings. The physical layout of the experimental setups and their indoor arrangement is depicted in Figure 3(b). The MPD’s positions in the *Z*-direction are adjusted using standard connecting rods, with heights accurately measured using a ruler. Figure 3(c) illustrates the arrangement of the red grids, green laser, and steel ruler. The inset provides a detailed illustration, demonstrating the precise alignment of the green laser with the red grids, with the green laser pointing to a determined reading on the steel ruler. The dimensions of the red laser grids can be adjusted based on the projection height. In this experiment, the total size of the grids was adjusted to 50 cm × 50 cm, with each square unit measuring as 1 cm in length. By traversing the green laser over each grid line, specific areas can be tested, and then by moving the grids, other areas can be subsequently tested.

### 2.4 Determination of the MPD initial position

**Figure 4:**
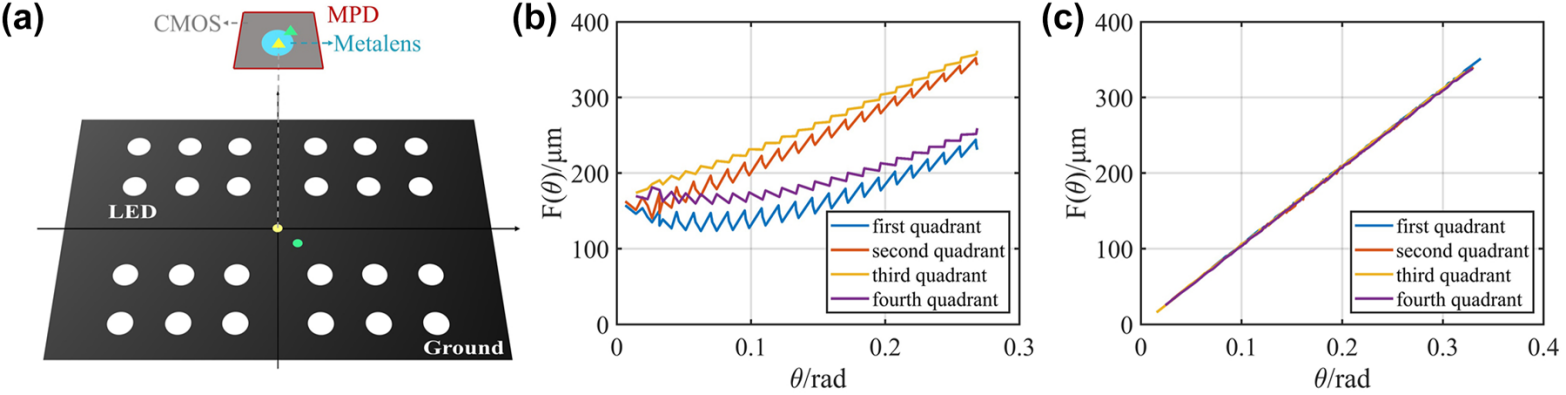
Determination of the MPD initial position. (a) Schematic diagram of determining the MPD initial position. The yellow and green circles on the ground represent the correct initial position and the coarsely determined initial position, respectively. The yellow and green triangles on CMOS represent the correct center of metalens and the coarsely determined center of metalens, respectively. (b) When the MPD position and the metalens center are not determined correctly, the curves of LED viewing angles *θ* and the image point distances *F*(*θ*) in four quadrants would be irregular. (c) When the MPD position and the metalens center are determined correctly, the curves of LED viewing angles *θ* and the image point distances *F*(*θ*) in four quadrants would completely overlap.

With the aforementioned positioning detection system, precise relative movement can be achieved. However, due to the certain relative distance between the MPD and the laser ruler, which is difficult to be directly and accurately measured, it is crucial to effectively determine the precise initial coordinate of the MPD for subsequent position calibration. Benefitting from the relationship between the imaging position and the LED viewing angle expressed in Eq. (3), the MPD initial coordinate can be obtained from imaging of multiple LEDs at different positions. We divided the experimental zone into four quadrants, with the center of the zone as the origin, and set LEDs symmetrically positioned in each quadrant, as shown in Figure 4(a). If the MPD initial position (shown as the blue triangle on the ground in Figure 4(a)) and the metalens center point (shown as the blue circle on CMOS in Figure 4(a)) are coarsely determined, the experimentally tested relation curves of LED viewing angles *θ* and the image point distances *F*(*θ*) in the four quadrants would be irregular, as shown in Figure 4(b). We traversed all points near the coarsely determined MPD initial position and the metalens center point and calculated the *θ* and *F*(*θ*) relation curves in four quadrants. An objective function was then set to characterize the total differences of these four curves, expressed as

(4) $$O=\underset{\theta }{\sum }\overset{4}{\underset{i=1}{\sum }}{\left(F_{i}\left(\theta \right)-\bar{F\left(\theta \right)}\right)}^{2},$$

where *F*_*i*_(*θ*) represents the image point distance corresponding to viewing angle *θ* of the *i*-th quadrant, and $\bar{F\left(\theta \right)}$ represents the average image point distance of the four quadrants. We ultimately determined the accurate MPD initial position and the metalens center, by finding the minimum value of objective function *O*. Based on the accurate positions, the calculated curves of *θ* and *F*(*θ*) would follow the *f* sin*θ* relation, and the four curves would completely overlap, as shown in Figure 4(c). The distance between the object and the LED can be precisely determined using the magnification ratio, as the imaging distance is fixed at *f*. The object distance equals the imaging distance multiplied by the magnification ratio. The detailed process for determining MPD initial position is shown in Supplementary Note S2.

The arrangement of the three beacon LEDs is also of importance to the positioning performances. In Supplementary Note S3, we conducted a detailed analysis of the relationship between the spacing of LEDs and positioning errors, ultimately determining that the positioning error is minimized when the spacing between three LEDs is set as 0.242 m.

### 2.5 Positioning performances of the MPD

**Figure 5:**
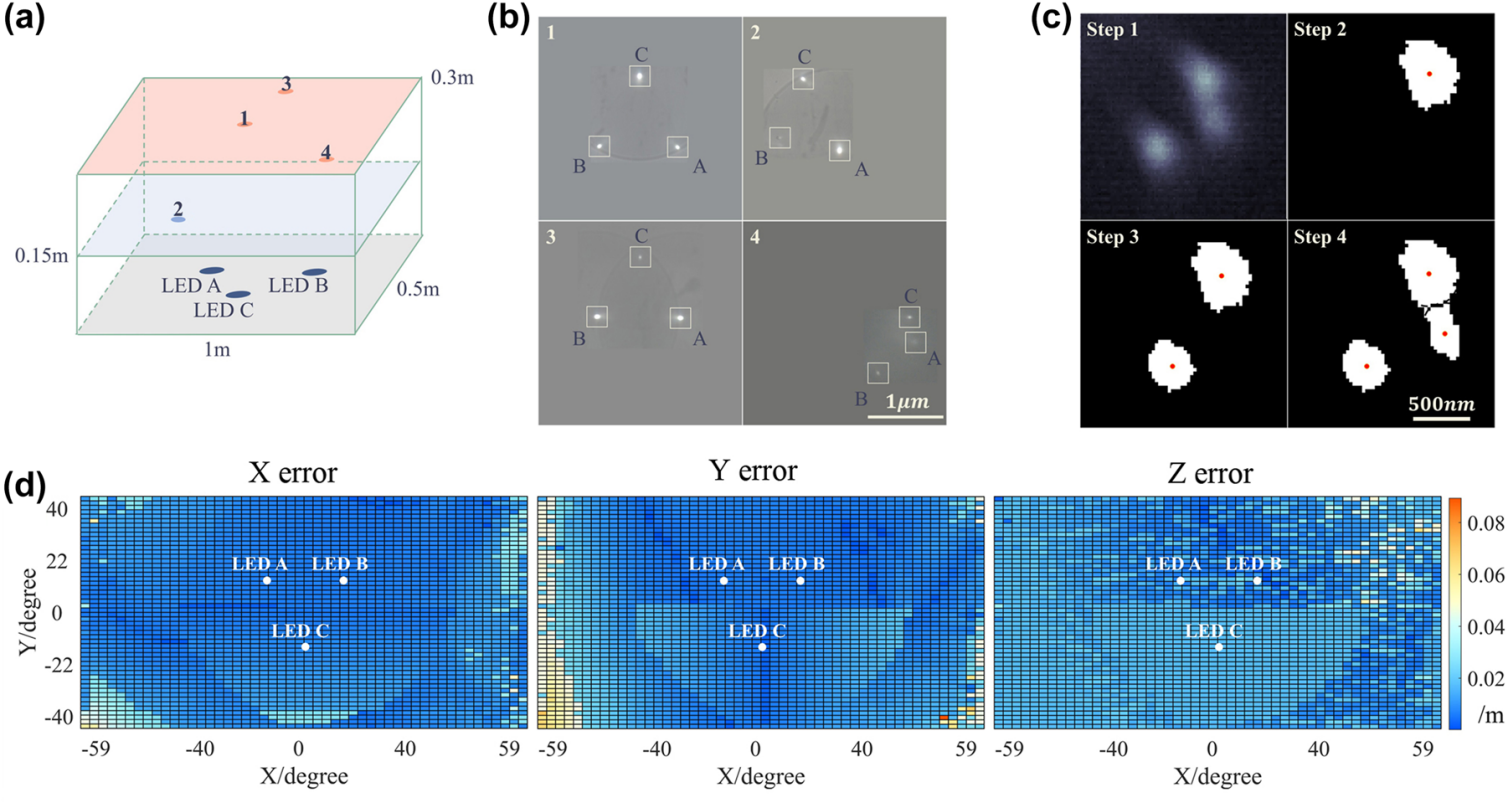
Positioning performance of the MPD. (a) Schematic of four MPD locations in the room and (b) the corresponding captured images. (c) Steps for determining the image positions of large viewing angle LEDs, the images of which are too weak to be analyzed directly. The red points represent the determined center of these LED images. (d) The analyzed positioning error distributions of the 0.3 m height plane, in *X*, *Y*, and *Z* directions, respectively. The relative positions of the three LEDs are marked in white. The experimental zone covers a viewing angle region over ±60° in *X* direction and ±40° in *Y* direction.

The MPD positioning performances are demonstrated by the positioning errors within the 1 m × 0.5 m experimental zone. Figure 5(a) shows the relative positions of four typical points in the experimental zone, three of which are located within the plane 0.3 m above the ground and one of which is located within a plane 0.15 m above the ground. The imaging results of 3 LEDs captured at these positions are shown in Figure 5(b), which indicate that at different positions, we would obtain different LEDs images distributions on CMOS. It can be seen from Figure 5(b) that when the LED viewing angle is small (position 1), the LED image would be relatively bright, making it easy to determine the image center. However, when the LED viewing angle is large enough (position 4, viewing angle near 60°), the imaging intensity would be too weak to directly determine the center.

To address the issue, we adopted a point-by-point threshold method for large viewing angle images. The purpose of this method is to binarize the image by applying different intensity thresholds, in order to facilitate the extraction of the LED images contours and centers. It is essential to set appropriate binarization thresholds for each LED image, as setting a threshold too high will result in an undetermined image contour, while setting it too low will introduce imaging noise into the contour.

Therefore, our method is referred to as the point-to-point threshold method. Figure 5(c) shows the steps of our method. Firstly, preprocessing of removing bad points from the image was conducted. Then the threshold was increased until a readable target LED image appears, shown as the step 2 in Figure 5(c). After determining the image center, this LED image area was covered up to avoid the determination of other LED images as the threshold increases. By repeating the above steps, all the three LED images contours and centers would be determined, shown as step 4 in Figure 5(c). The detailed process of this method is shown in Supplementary Note S4.

Positioning errors were calculated by comparing experimentally obtained position coordinates with ground truth position coordinates at each testing point. A total of 2,601 (51 × 51) positions were tested by the MPD on the 0.3 m height plane within the 1 m × 0.5 m experimental zone. This zone covers a wide-angle region spanning over ±60° in the *X* direction and ±40° in the *Y* direction, with sampling intervals of 2 cm and 1 cm, respectively. The calculated positioning errors in the *X*, *Y*, and *Z* directions are 0.0095 m, 0.0116 m, and 0.015 m, respectively. This indicates a positioning accuracy approaching the millimeter-level across the wide-angle area. However, due to inevitable operational errors inherent in testing thousands of positions, certain points may exhibit significant errors. When averaging positioning accuracy, these points are included, potentially skewing the overall assessment. To address this, we also calculated the positioning accuracy for 90 % and 80 % of the positions in the region, detailed in Table 1. These results demonstrate that positioning accuracies in the majority of the wide-angle region have indeed reached millimeter-level precision. From the positioning accuracy results, it can be observed that the method exhibits slightly lower accuracy in the *z* direction compared to the *x* and *y* directions. From the perspective of the imaging model shown in Figure 1(b), this discrepancy arises because the movements in the *x* and *y* directions can be directly reflected by the LED imaging positions’ changes on CMOS. However, movements in the *z* direction can only be indirectly reflected in the LED imaging positions’ changes, through a geometric projection, resulting in additional positioning errors.

**Table 1:** Average positioning accuracies for different percentages of area.

Area percentages	*X* error (m)	*Y* error (m)	*Z* error (m)
100 %	0.0095	0.0116	0.0142
90 %	0.0078	0.0091	0.0129
80 %	0.0073	0.0080	0.0124

The MPD’s imaging performance is primarily influenced by the viewing angle rather than the object distance. As a result, the positioning accuracy of the MPD is mainly determined by the viewing angle. Therefore, the millimeter precision positioning performance of the MPD remains applicable to larger areas, provided that the viewing angle is smaller than ±60°. This conclusion is experimentally supported by positioning accuracy results across different height planes, as depicted in Supplementary Note S5.

## 3 Discussion and conclusion

Our method can be expanded to not only achieve (*x*, *y*, *z*) three-dimensional coordinate positioning but also determine the attitude angles (*ψ*_*x*_, *ψ*_*y*_) of the MPD device. However, in this case, five beacon LEDs are necessary. By establishing imaging relationships for these LEDs, five independent geometric equations can be formulated, thus enabling the determination of all position and orientation angle information. The detailed derivation is provided in Supplementary Note S6. Furthermore, our approach demonstrates excellent scalability in terms of workspace expansion. By simply increasing the number of beacon LEDs, the working range can be easily extended to hundreds of meters. This enables our scheme to be applicable not only for positioning within single rooms but also for scenarios such as indoor parking lots, large shopping malls, and warehouses. The position information can be encoded into the on–off states of LEDs and captured by the camera [45]. Detailed analysis regarding the scalability of the workspace expansion is provided in Supplementary Note S7. When the viewing angle of LED gets larger, the imaging intensity decreases, which will lead to the reduction of positioning accuracy. One effective way to improve the large viewing angle imaging efficiency involves topological optimization, which targets the imaging efficiency as the optimization objective, and use the structure shapes as optimization parameters [46]. Through the optimization process, it is possible to achieve free-form structure units or nonperiodic arranging units with high modulation efficiency for large viewing angle imaging.

Compared to traditional lens positioning devices, the advantages of our metalens-based device are reflected in the following aspects. First, traditional lenses are typically bulk lenses with curved surfaces. In contrast, metalenses are designed and fabricated on a flat surface, with an ultra-thin thickness, making our device more compact. Second, thanks to the unique polarization characteristics of our geometric phase metalens, the imaging contrast of beacon LEDs can be effectively enhanced through polarization filtering when there is significant noise interference, thereby improving the positioning accuracy. Third, the metalens we designed possesses the capability for wide-angle imaging, which is difficult to achieve with a single traditional bulk lens (see Supplementary Note S8 for detailed analysis). Therefore, our positioning device based on the wide-angle metalens offers a much larger working range. In addition, the metasurface unit structure provides a vast space for parameter design and optimization, which offers a pathway to achieve more complex functions, such as high-efficiency wide-angle imaging.

In summary, utilizing a metalens-integrated camera, we have achieved high-precision positioning in a wide-angle area by imaging beacon LEDs. The quadratic phase metalens ensures accurate imaging of beacon LEDs within a 120° viewing angle range, unaffected by external signals. Consequently, our method achieves millimeter-level positioning accuracy across most parts of the wide-angle experimental region. Moreover, we have introduced a point-by-point threshold binarization method for low-contrast images to tackle the challenge of determining the imaging positions of low intensity LED images, caused by large viewing angle. This approach ensures high positioning accuracy within 120° field of view, ultimately delivering millimeter-level accuracy for the majority of positions. Beyond its high-precision positioning capabilities, the ultra-compact size of our MPD makes it highly suitable for integration into camera modules of smartphones, drones, and other devices, presenting a novel technical solution for future high-performance indoor communication, IoT, and related fields.

## 4 Experimental section

Metalens fabrication: step 1, deposit an undoped layer of silicon nitride (Si_3_N_4_) with thickness of 800 nm on the fused-silica substrate (SiO_2_) using plasma-enhanced chemical vapor deposition (PECVD) method. Step 2, spin coat 200 nm PMMA A4 resist film onto the surface of the material to be processed, and bake at 170 °C for 5 min. Step 3, a 42 nm thick layer of water-soluble conductive polymer (AR-PC 5090) was next spin coated on the resist on which the metalens structure pattern was written by using an E-beam writer (Elionix ELS-F125). The conductive polymer was then dissolved in water, and resist was developed in a resist developer solution. Step 4, an electron beam evaporated chromium layer was used to reverse the generated pattern with a lift-off process and was then used as a hard mask for dry etching the silicon nitride layer. Step 5, the dry etching was performed in a mixture of CHF3 and SF6 plasmas using an inductively coupled plasma reactive ion etching process (Oxford Instruments, PlasmaPro 100 Cobra300). Step 6, the chromium layer was removed by a stripping solution (Ce(NH_4_)_2_(NO_3_)_6_).

## Supporting Information

See Supplementary 1 for supporting content.

## Supplementary Material

Supplementary Material Details
