# Supplementary material for: Millimeter-precision positioning for wide-angle indoor area enabled by metalens-integrated camera
Source: Nanophotonics. 2024 Aug 29;13(22):4101–10. doi: 10.1515/nanoph-2024-0277 (PMC11501052; doi:10.1515/nanoph-2024-0277)
Supplement: Supplementary file 1 — Supplementary Material Details [file j_nanoph-2024-0277_suppl_001.docx]

**Supplementary Materials to**

Millimeter-precision positioning for wide-angle indoor area enabled by metalens-integrated camera

Muyang Li,^1^ Yue Wu,^1^ Haobai Li,^1^ Zi-Wen Zhou,^1^ Yanxiang Zhang,^1^ Zhongyi Yuan,^1^ Zaichen Zhang^1,2,*^ and Ji Chen^1,2,*^

^1^National Mobile Communications Research Laboratory, School of Information Science and Engineering, Frontiers Science Center for Mobile Information Communication and Security, Southeast University, Nanjing 210096, China.

^2^Purple Mountain Laboratories, Nanjing 211111, China.

*Corresponding authors: Ji Chen, e-mail: [jichen@seu.edu.cn](mailto:jichen@seu.edu.cn); Zaichen Zhang, E-mail: [zczhang@seu.edu.cn](mailto:zczhang@seu.edu.cn).

Supplementary material contains Note S1-S8, Fig. S1-S8, and Table S1-S2.

**Table S1 Performance comparisons of positioning technologies**

| **Representative Works** | **Device** | **Dimension** | **Accuracy** | **FOV** | **Working band** | **Device size** |
| --- | --- | --- | --- | --- | --- | --- |
| **This work** | **Metalens** | **3** | **0.009m** | **120°** | **Visible light** | **3cm×3cm**  **×0.3cm** |
| *Nat. Commun*. 15, 2844 (2024).  From Northeastern University, USA | RF board | 1 | 0.16m | / | 836MHz radio | 20cm×20cm  ×1cm |
| *Nat. Commun.* 14, 1035 (2023).  From Tsinghua University, China | Metalens | 1 | 0.003m | / | Visible light | 5cm×20cm  ×10cm |
| *Adv. Photon*. 1, 3 (2019).  From Friedrich Schiller University Jena, Germany | Metalens | 1 | 0.01m | / | Visible light | 10cm×10cm  ×1cm |
| *J. Lightwave Technol*. 41, 5564 (2023).  From Fujian Normal University, China | PD | 2 | 0.03m | 80° | Visible light | 5cm×10cm  ×5cm |
| *IEEE Trans. Instrum. Meas.* 70, 1 (2021).  From Massey University, New Zealand | PD+IMU | 2 | 0.012m | 60° | Visible light | 12cm×12cm  ×3cm |
| *J. Lightwave Technol*. 32, 3306 (2014).  From University of South Australia, Australia | PD+IMU | 3 | 0.25m | 50° | Visible light | Not Integrated |
| *IEEE Photonics J*. 12, 6 (2020).  From Guangxi University, China | Camera | 2 | 0.025m | 40° | Visible light | 5cm×5cm  ×4cm |
| *Opt. Lett*. 48, 6468 (2023).  From Information Engineering University, China | Camera | 2 | 0.025m | 60° | Visible light | 5cm×20cm  ×10cm |
| *IEEE Photonics J.* 12, 1 (2020).  From Hong Kong University of Science and Technology, China | Camera | 3 | 0.082m | 50° | Visible light | 20cm×30cm  ×20cm |
| *Opt. Express*. 29, 4582 (2021).  From Edith Cowan University, Australia | PD | 3 | 0.054m | 80° | 1550nm IR | Not Integrated |
| *IEEE Trans. Instrum. Meas.* 70, 1 (2021).  From Politecnico of Torino, Italy | UWB board | 2 | 0.1m | / | 7.25GHz radio | 18cm×20cm  ×8cm |
| *IEEE Trans. Ultrason., Ferroelect., Freq. Contr.* 69, 1469 (2022).  From Tianjin University, China | MEMS | 3 | 0.0346m | / | 25 kHz ultrasonic | 10cm×10cm  ×3cm |
| *IEEE Trans. Instrum. Meas.* 69, 1962 (2020).  From University of Science and Technology of China, China | MEMS | 3 | 0.4m | / | 15–22 kHz ultrasonic | 15cm×20cm  ×10cm |

Note:

**“/”** means “Not measured”; **PD:** photodiode; **IMU:** inertial measurement unit; **IR:** infrared ray;

**UWB:** ultrawideband; **MEMS:** micro-electro mechanical system; **RF:** radio frequency; **FOV:** field of view

**Note S1 Polarization filtering based on geometric phase metasurface**

We utilize geometric phase for metalens design to ensure that the MPD device maintains optimal performance in environment with high noise level. In scenarios where ambient light significantly interferes with the imaging of beacon LEDs, the polarization filtering based on geometric phase metasurfaces would be very useful. Figure S1(a) shows the polarization filtering scheme under the condition of noise interference. The light emitted from beacon LED is set to be left-handed circularly polarized (LCP), and the imaging of the LED would be modulated as right-handed circularly polarized (RCP) by the metasurface. At this point, the RCP polarizer behind the metasurface would effectively eliminate extraneous noise, while ensure the passing through of LED light. Figure S1(b) and (c) show the captured LED imaging, with and without polarization filtering, respectively. It can be observed that with the polarization filtering, the imaging signal-to-noise is obviously enhanced, thereby contributing to the improvement of positioning accuracy.


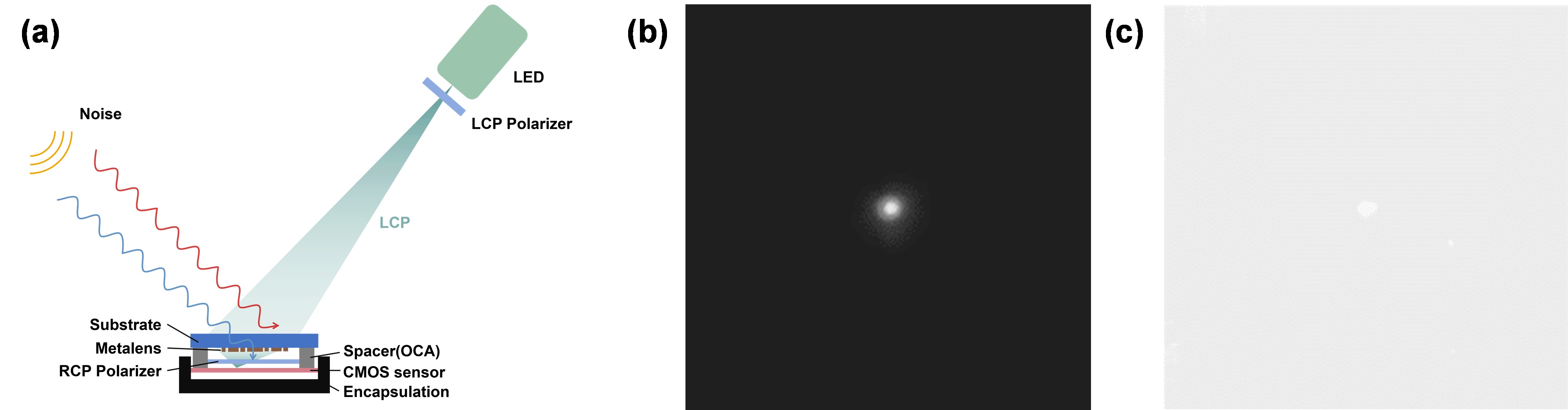


**Figure S1:** Polarization filtering based on geometric phase metalens. (a) Schematic diagram of polarization filtering. LED imaging result (b) with and (c) without RCP polarizer filtering.

**Note S2 Detailed process for determination of the MPD initial position**

The schematic diagram Fig. S2(a) illustrates the necessity of determining the location of the MPD center and MPD position. Firstly, when manufacturing the metalens on the substrate using microfabrication technology, the accurate position of the structure on the substrate is unknown. Secondly, during the MPD integration process, the relative position between the metalens and the CMOS is also unknown. Figure S2(a) shows the misalignment of this position. According to Section 2.4 of the main text, in order to conduct precise positioning experiments, we need to know the position where the center of the metalens projects on the CMOS (referred to as the MPD center), as shown by the yellow triangle in Fig. S2(a), and the position where the center of the metalens projects on the ground (referred to as the MPD position), as shown by the yellow circle in Fig. S2(a).

As mentioned in the main text, we divided the experimental area into four quadrants based on the position of the MPD, and placed the LED in each quadrant. We calculated the relationship between the LED incidence angle and the image point distances in each quadrant, and obtained four curves.

The flow chart of the algorithm of determining the location of the MPD center and MPD position is shown in Fig. S2(b). We first traverse the Y dimension of the MPD center point and the Y dimension of the MPD position that needs to be determined, and find the Y direction position of the point with the highest similarity in the curves of the first and fourth quadrants, as well as the second and third quadrants. On the basis of determining the Y position, traverse the X dimension of the MPD center point and the X dimension of the MPD position to find the X direction position of the point with the highest similarity in the first and second quadrants, as well as the third and fourth quadrants. The MPD center and MPD position with the highest degree of similarity in each quadrant curve can be considered accurate.

We use the sampling point method to characterize the differences between these curves, and the objective function for optimizing is

$$\begin{aligned} S^{2}=\frac{1}{n}\sum_{i=1}^{n} (a_{i}-b_{i})^{2}\#\left( S2.1 \right) \end{aligned}$$

While $(a_{1},a_{2},\ldots,a_{n})$ and $(b_{1},b_{2},\ldots,b_{n})$ are the corresponding sampling point values of different curves.

Figure S2(c) shows the curve of the incident angle and the image point distance when the assumed MPD center and MPD position are in the wrong position. Figure S2(d) shows the relationship curve during the algorithm traversal process, when the assumed Y coordinates of the MPD center and MPD position are optimized. Figure S2(e) shows the traversal algorithm result and the assumed MPD center and MPD position are in the correct position, the relationship curve of the incident angle and the image point distance.


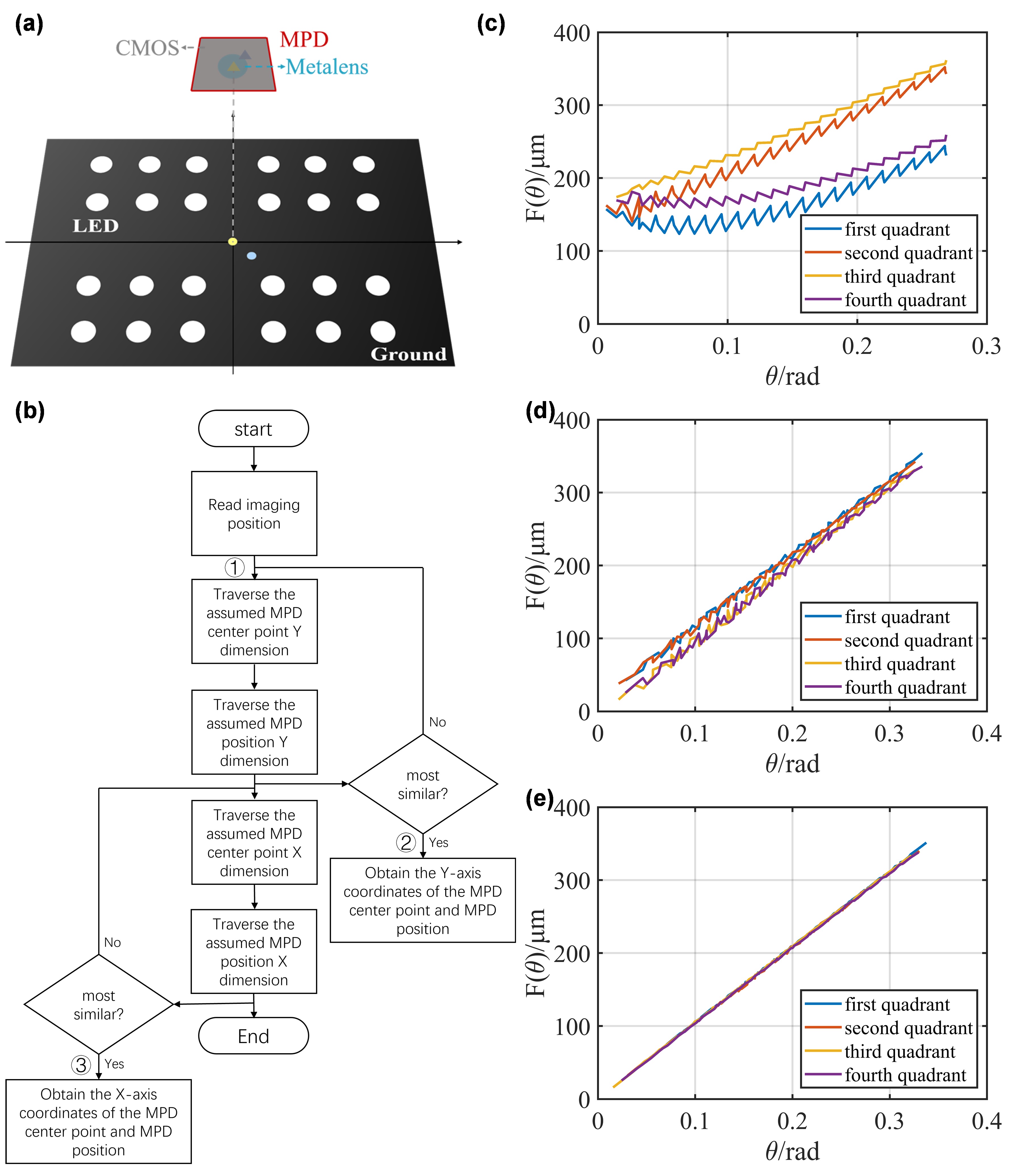


**Figure S2:** Experimental model and algorithm flow chart of accurate measurement method for MPD center and MPD position. (a) Architecture diagram of precision measurement scheme for MPD center and MPD position. The yellow circle represents the correct MPD position, the yellow triangle represents the correct MPD center, the blue circle represents the incorrect MPD position, and the blue triangle represents the incorrect MPD center. (b) Flow chart of precise measurement algorithm for MPD center and MPD position. (c) The curve of the incident angle and the image point distance when the assumed MPD center and MPD position are in the wrong position. Draw in the four quadrants centered around the assumed MPD position. Corresponding to position 1 in the flowchart in (b). (d) During the algorithm traversal process, the curve of the incident angle and the image point distance is obtained when the assumed Y coordinates of the MPD center and MPD position are the same as the correct position. Corresponding to position 2 in the flowchart in (b). (e) When the traversal algorithm is completed and the assumed MPD center and MPD position are in the correct position, the curve of the incident angle and the image point distance. Corresponding to position 3 in the flowchart in (b).

**Note S3 Optimization for the Distance between Three LEDs**

As the reference beacon for positioning measurement, the position distribution of the three LEDs can affect the MPD average positioning accuracy. Therefore, it is necessary to explore the relationship between the distribution of the three LEDs and the average positioning accuracy before implementing experiment, to achieve the highest average positioning accuracy under model conditions.

The relationship between the imaging position on CMOS $X_{i}$ and the light incidence angle $\theta_{i}$ of quadratic metalens can be expressed as $fsin\theta_{i}$. Given three LED ground coordinates as $\left( a_{1},b_{1} \right),\left( a_{2},b_{2} \right),(a_{3},b_{3})$, after obtaining the position vectors $X_{1},X_{2},X_{3}$ of the three LED imaging points on CMOS, the equation for the three-dimensional coordinates of MPD $(x, y, H)$ can be expressed as follows:

$$\begin{aligned} \left\{ \begin{matrix} {X_{1}}^{2}=f^{2}\cdot\frac{\left( x-a_{1} \right)^{2}+\left( y-b_{1} \right)^{2}}{H^{2}+\left( x-a_{1} \right)^{2}+\left( y-b_{1} \right)^{2}} \\ {X_{2}}^{2}=f^{2}\cdot\frac{\left( x-a_{2} \right)^{2}+\left( y-b_{2} \right)^{2}}{H^{2}+\left( x-a_{2} \right)^{2}+\left( y-b_{2} \right)^{2}} \\ {X_{3}}^{2}=f^{2}\cdot\frac{\left( x-a_{3} \right)^{2}+\left( y-b_{3} \right)^{2}}{H^{2}+\left( x-a_{3} \right)^{2}+\left( y-b_{3} \right)^{2}} \end{matrix} \right.,\#\left( S3.1 \right) \end{aligned}$$

where $f$ represents the focus length of metalens. By solving this equation, four possible coefficient solutions can be obtained, the $x$ part of the four solutions $\left( x_{1}, y_{1},H_{1} \right),\left( x_{2}, y_{2},H_{2} \right),\left( x_{3}, y_{3},H_{3} \right),\left( x_{4}, y_{4},H_{4} \right)$ are as follows:

$$\begin{aligned} x_{1}=\frac{\sqrt{\left( a_{1}^{2}+b_{1}^{2} \right)\left( a_{2}^{2}+b_{2}^{2} \right)\left( a_{3}^{2}+b_{3}^{2} \right)}}{8}-\frac{M_{1}}{N},\#\left( S3.2 \right) \end{aligned}$$

$$\begin{aligned} x_{2}=\frac{\sqrt{\left( a_{1}^{2}+b_{1}^{2} \right)\left( a_{2}^{2}+b_{2}^{2} \right)\left( a_{3}^{2}+b_{3}^{2} \right)}}{8}+\frac{M_{1}}{N},\#\left( S3.3 \right) \end{aligned}$$

$$\begin{aligned} x_{3}=\frac{\sqrt{\left( a_{1}^{2}+b_{1}^{2} \right)\left( a_{2}^{2}+b_{2}^{2} \right)\left( a_{3}^{2}+b_{3}^{2} \right)}}{8}-\frac{M_{2}}{N},\#\left( S3.4 \right) \end{aligned}$$

$$\begin{aligned} x_{4}=\frac{\sqrt{\left( a_{1}^{2}+b_{1}^{2} \right)\left( a_{2}^{2}+b_{2}^{2} \right)\left( a_{3}^{2}+b_{3}^{2} \right)}}{8}+\frac{M_{2}}{N},\#\left( S3.5 \right) \end{aligned}$$

where $M_{1},M_{2},N$ represent:

$$\begin{aligned} N=\left( {X_{2}}^{2}-f^{2} \right)\left( a_{1}-b_{1} \right)\left( 2{X_{1}}^{2}-2f^{2} \right)\left( {X_{1}}^{4}f^{4}-2{X_{1}}^{2}{X_{2}}^{2}f^{4}+{X_{2}}^{4}f^{4} \right),\#\left( S3.6 \right) \end{aligned}$$

$$M_{1}=\left( {X_{1}}^{2}f^{2}-{X_{2}}^{2}f^{2} \right)\left( {X_{1}}^{2}{a_{1}}^{2}f^{6}-2{X_{1}}^{4}{X_{2}}^{4}{b_{1}}^{2}-2{X_{1}}^{4}{X_{2}}^{4}{a_{1}}^{2}-{X_{1}}^{4}{a_{1}}^{2}f^{4}+{X_{2}}^{2}{a_{1}}^{2}f^{6}-{X_{2}}^{4}{a_{1}}^{2}f^{4}+{X_{1}}^{2}{b_{1}}^{2}f^{6}-{X_{1}}^{4}{b_{1}}^{2}f^{4}+{X_{2}}^{2}{b_{1}}^{2}f^{6}-{X_{2}}^{4}{b_{1}}^{2}f^{4}+2X_{1}X_{2}{a_{1}}^{2}\sqrt{\left( X_{1}+f \right)^{3}\left( X_{2}+f \right)^{3}\left( X_{1}-f \right)^{3}\left( X_{2}-f \right)^{3}}+2X_{1}X_{2}{b_{1}}^{2}\sqrt{\left( X_{1}+f \right)^{3}\left( X_{2}+f \right)^{3}\left( X_{1}-f \right)^{3}\left( X_{2}-f \right)^{3}}-4{X_{1}}^{2}{X_{2}}^{2}{a_{1}}^{2}f^{4}+3{X_{1}}^{2}{X_{2}}^{4}{a_{1}}^{2}f^{2}+3{X_{1}}^{4}{X_{2}}^{2}{a_{1}}^{2}f^{2}-4{X_{1}}^{2}{X_{2}}^{2}{b_{1}}^{2}f^{4}+3{X_{1}}^{2}{X_{2}}^{4}{b_{1}}^{2}f^{2}+3{X_{1}}^{4}{X_{2}}^{2}{b_{1}}^{2}f^{2}+4{X_{1}}^{4}{X_{2}}^{4}a_{1}b_{1}-2{X_{1}}^{2}a_{1}b_{1}f^{6}+2{X_{1}}^{4}a_{1}b_{1}f^{4}-2{X_{2}}^{2}a_{1}b_{1}f^{6}+2{X_{2}}^{4}a_{1}b_{1}f^{4}-4X_{1}X_{2}a_{1}b_{1}\sqrt{\left( X_{1}+f \right)^{3}\left( X_{2}+f \right)^{3}\left( X_{1}-f \right)^{3}\left( X_{2}-f \right)^{3}}+8{X_{1}}^{2}{X_{2}}^{2}a_{1}b_{1}f^{4}-6{X_{1}}^{2}{X_{2}}^{4}a_{1}b_{1}f^{2}-6{X_{1}}^{4}{X_{2}}^{2}a_{1}b_{1}f^{2} \right),$$

$$\begin{aligned} \#\left( S3.7 \right) \end{aligned}$$

$$M_{2}=({X_{1}}^{2}f^{2}-{X_{2}}^{2}f^{2})(2{X_{1}}^{4}{X_{2}}^{4}{a_{1}}^{2}+2{X_{1}}^{4}{X_{2}}^{4}{b_{1}}^{2}-{X_{1}}^{2}{a_{1}}^{2}f^{6}+{X_{1}}^{4}{a_{1}}^{2}f^{4}-{X_{2}}^{2}{a_{1}}^{2}f^{6}+{X_{2}}^{4}{a_{1}}^{2}f^{4}-{X_{1}}^{2}{b_{1}}^{2}f^{6}+{X_{1}}^{4}{b_{1}}^{2}f^{4}-{X_{2}}^{2}{b_{1}}^{2}f^{6}+{X_{2}}^{4}{b_{1}}^{2}f^{4}+2X_{1}X_{2}{a_{1}}^{2}\sqrt{{(X_{1}+f)}^{3}{(X_{2}+f)}^{3}{(X_{1}-f)}^{3}{(X_{2}-f)}^{3}}+2X_{1}X_{2}{b_{1}}^{2}\sqrt{{(X_{1}+f)}^{3}{(X_{2}+f)}^{3}{(X_{1}-f)}^{3}{(X_{2}-f)}^{3}}+4{X_{1}}^{2}{X_{2}}^{2}{a_{1}}^{2}f^{4}-3{X_{1}}^{2}{X_{2}}^{4}{a_{1}}^{2}f^{2}-3{X_{1}}^{4}{X_{2}}^{2}{a_{1}}^{2}f^{2}+4{X_{1}}^{2}{X_{2}}^{2}{b_{1}}^{2}f^{4}-3{X_{1}}^{2}{X_{2}}^{4}{b_{1}}^{2}f^{2}-3{X_{1}}^{4}{X_{2}}^{2}{b_{1}}^{2}f^{2}-4{X_{1}}^{4}{X_{2}}^{4}a_{1}b_{1}+2{X_{1}}^{2}a_{1}b_{1}f^{6}-2{X_{1}}^{4}a_{1}b_{1}f^{4}+2{X_{2}}^{2}a_{1}b_{1}f^{6}-2{X_{2}}^{4}a_{1}b_{1}f^{4}-4X_{1}X_{2}a_{1}b_{1}\sqrt{{(X_{1}+f)}^{3}{(X_{2}+f)}^{3}{(X_{1}-f)}^{3}{(X_{2}-f)}^{3}}-8{X_{1}}^{2}{X_{2}}^{2}a_{1}b_{1}f^{4}+6{X_{1}}^{2}{X_{2}}^{4}a_{1}b_{1}f^{2}+6{X_{1}}^{4}{X_{2}}^{2}a_{1}b_{1}f^{2}).$$

$$\begin{aligned} \#\left( S3.8 \right) \end{aligned}$$

Among these four solutions $\left( x_{1}, y_{1},H_{1} \right),\left( x_{2}, y_{2},H_{2} \right),\left( x_{3}, y_{3},H_{3} \right),\left( x_{4}, y_{4},H_{4} \right)$, according to the objective constraints of model parameter $H$, a unique solution that meets the constraints can be selected. Restrictions can be expressed as:

$$\begin{aligned} \underset{i}{\mathrm{argmin}} \left\{ H_{i}-H_{real} \right\}, H>0, H\in\boldsymbol{R}\mathbf{,} i\in\left[ 1,4 \right]\#\left( S3.9 \right) \end{aligned}$$

Record the $x$ part of the unique solution as $x_{o}$:

$$\begin{aligned} x_{o}=x_{1}=\frac{\sqrt{\left( a_{1}^{2}+b_{1}^{2} \right)\left( a_{2}^{2}+b_{2}^{2} \right)\left( a_{3}^{2}+b_{3}^{2} \right)}}{8}-\frac{M_{1}}{N}.\#\left( S3.10 \right) \end{aligned}$$

Then according to the law of sines, $\sqrt{a_{i}^{2}+b_{i}^{2}}$ as projection length is directly proportional to incident angle $\theta_{i}$. So $\sqrt{a_{i}^{2}+b_{i}^{2}}$ can be represented as a proportional substitute for $\theta_{i}$:

$$\begin{aligned} \sqrt{a_{i}^{2}+b_{i}^{2}}=k_{i}\theta_{i}\#\left( S3.11 \right) \end{aligned}$$

$$\begin{aligned} x_{o}=\frac{{k_{1}k_{2}k_{3}\theta}_{1}\theta_{2}\theta_{3}}{8}-g\left( \theta\right)\#\left( S3.12 \right) \end{aligned}$$

$g\left( \theta\right)=\frac{M_{1}}{N}$ is a higher-order function of variables ${\theta_{1},\theta}_{2},\theta_{3}$.

Considering the incident angle $\theta_{i}$ is small, and for simplicity set the coefficient to 1, the coefficient solution can be simplified as

$$\begin{aligned} x_{o}=\theta_{1}\theta_{2}\theta_{3} , i=1,2,3\#\left( S3.13 \right) \end{aligned}$$

Then according to the error transfer formula (mean square error)

$$for X=uvw$$

$$\begin{aligned} M.S.E \sigma_{X}^{2}=\sigma_{u}^{2}\cdot v^{2}\cdot w^{2}+\sigma_{v}^{2}\cdot u^{2}\cdot w^{2}+\sigma_{w}^{2}\cdot u^{2}\cdot w^{2}\#\left( S3.14 \right) \end{aligned}$$

When there is a reading error $\sigma_{i}$ under $i$th incident angle. The position error of the solved MPD is

$$\begin{aligned} \sigma_{x}^{2}=\sigma_{1}^{2}\cdot\theta_{2}^{2}\cdot\theta_{3}^{2}+\sigma_{2}^{2}\cdot\theta_{1}^{2}\cdot\theta_{3}^{2}+\sigma_{3}^{2}\cdot\theta_{1}^{2}\cdot\theta_{2}^{2}\#\left( S3.15 \right) \end{aligned}$$

According to the relationship of the imaging position of quadratic metalens shown in Fig. 2(c) of the main text, the reading error $\sigma_{i}$ and the incidence angle $\theta_{i}$ are directly proportional. So $\sigma_{i}$ in the MPD position error formula can be replace accordingly by $\theta_{i}$. Then the MPD positioning error formula is

$$\begin{aligned} \sigma_{x}=\sqrt{3}\theta_{1}\theta_{2}\theta_{3}\#\left( S3.16 \right) \end{aligned}$$

When three LEDs are distributed in an equilateral triangle centered on the MPD, the final MPD positioning error formula can be expressed as

$$\begin{aligned} \sigma_{x}=\sqrt{3}\left( arctan(\frac{r}{H}) \right)^{3}\#\left( S3.17 \right) \end{aligned}$$

where $r$ is the LED spacing, the distance between LED and the projection point of MPD projected onto the LED plane**,** $H$ is the height of the model.

Figure S3(a) shows under the model height parameter of 0.3m, the relationship between LED spacing and average positioning error of MPD when three LEDs are distributed in equilateral triangles. The LED spacing distribution used in the experiment is close to the optimized result. Figures S3(b)-(c) shows the relationship between the optimal LED spacing and the model parameters. By adjusting the maximum incident angle parameter from the edge of the model to MPD, we can draw the incident angle from the optimal LED distribution position to MPD. From Fig. S3(b), it can be seen that the maximum incident angle from the model edge to MPD shows the same trend as the incident angle from optimal LED position to MPD. Figure S3(c) also shows that the two incident angles are approximately linearly correlated. In summary, there is a definite relationship between the model parameters and the optimal LED spacing. This result can provide reference for LED layout in different scene models.


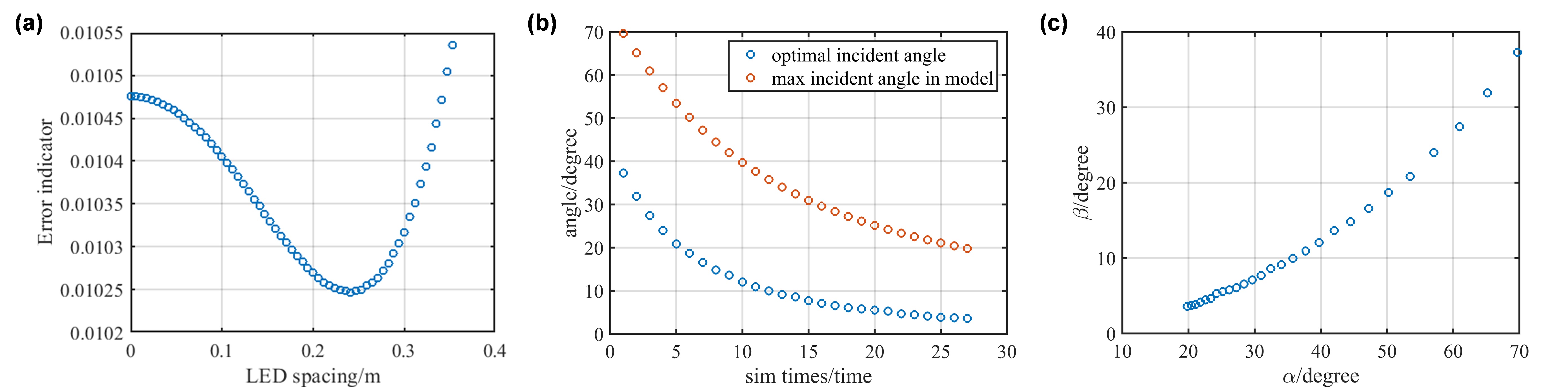


**Figure S3:** The relationship between the optimal LED spacing and the model parameters. (a) Under the model height of 0.3 meters, the relationship between LED spacing and average positioning error of MPD when three LEDs are distributed in equilateral triangle. (b) When the times of simulation increase, the trend of the maximum incident angle from the model edge to MPD and the same trend of the incident angle from optimal LED position to MPD. (c) When the maximum incident angle $\alpha$ from the model edge to MPD is the independent variable, the linearly correlated trend of the dependent variable the incident angle $\beta$ from optimal LED position to MPD.

**Note S4 Details of point-by-point threshold algorithm steps for imaging results at different positions**

At different positions of the model shown in Fig. S4(a), the MPD imaging image is shown in the first column on the left side of Fig. S4(c), and the number of each row in Fig. S4(c) corresponds to the position number on Fig. S4(a). Position 5 is located in the center of the model, so the imaging brightness of the three LEDs is the same. And the distribution of LED image in position 5 is similar to the actual LED distribution in an equilateral triangle. Positions 4 and 6 are located on symmetrical sides, while the distance among MPD and the three LEDs is not the same. It can be seen that the brightness and position distribution of the image have changed. This can be attributed to the characteristics of quadratic metalens. According to Eq. (3) in the main text, the imaging offset of the quadratic phase follows a sine relationship as $fsin\theta_{i}$, and the first derivative of the imaging offset follows a cosine relationship. Therefore, as the incident angle gets greater, the imaging offset increases slower as the incident angle changes. This explains the deformation of the position distribution of the image. This phenomenon can also be observed in positions 9, 2, 7, and 3, where it can be clearly seen that the offset distance of imaging points with high angle incidence is significantly shorter, resulting in severe compression of the triangular image distribution.

There is also a significant difference in image brightness between different incident angles in the wide-angle LED positioning experiment. Therefore, we introduced a point-by-point threshold algorithm in the wide-angle imaging center extraction step of the positioning experiment to extract and recognize target points.

The idea of the pointwise thresholding method is to better segment the image and extract points by selecting an appropriate threshold for each point that needs to be recognized. Our algorithm process is shown in Fig. S4(b). Firstly, we perform preprocessing steps such as removing bad points from the image, and then continuously increase the threshold until a readable target point appears. After determining the number of target points, read and record the position of the target points, masking the read points to avoid affecting the reading of other points. Afterwards, we continue to increase the threshold and repeat the above steps until the positions of all three target points are extracted, or the threshold is raised to the upper limit of the image. Then, based on the geometric relationship of the LEDs, determine the correspondence between the extracted target point and the LEDs.

The main text shows an example of point-by-point threshold algorithm result, which locates at point 1, the edge of the model. The schematic diagram of more point-by-point threshold results at different positions is shown in Fig. S4(c), which shows the results generated by the point-by-point threshold algorithm at different angles of the acquisition model, with each row representing the generated results at a certain angle of the acquisition model. The selected positions are distributed at various positions in the model, as shown in Fig. S4(a), so the results are representativeness, indicating that the point-by-point threshold point-by-point threshold algorithm is suitable for image processing under large angle imaging conditions.


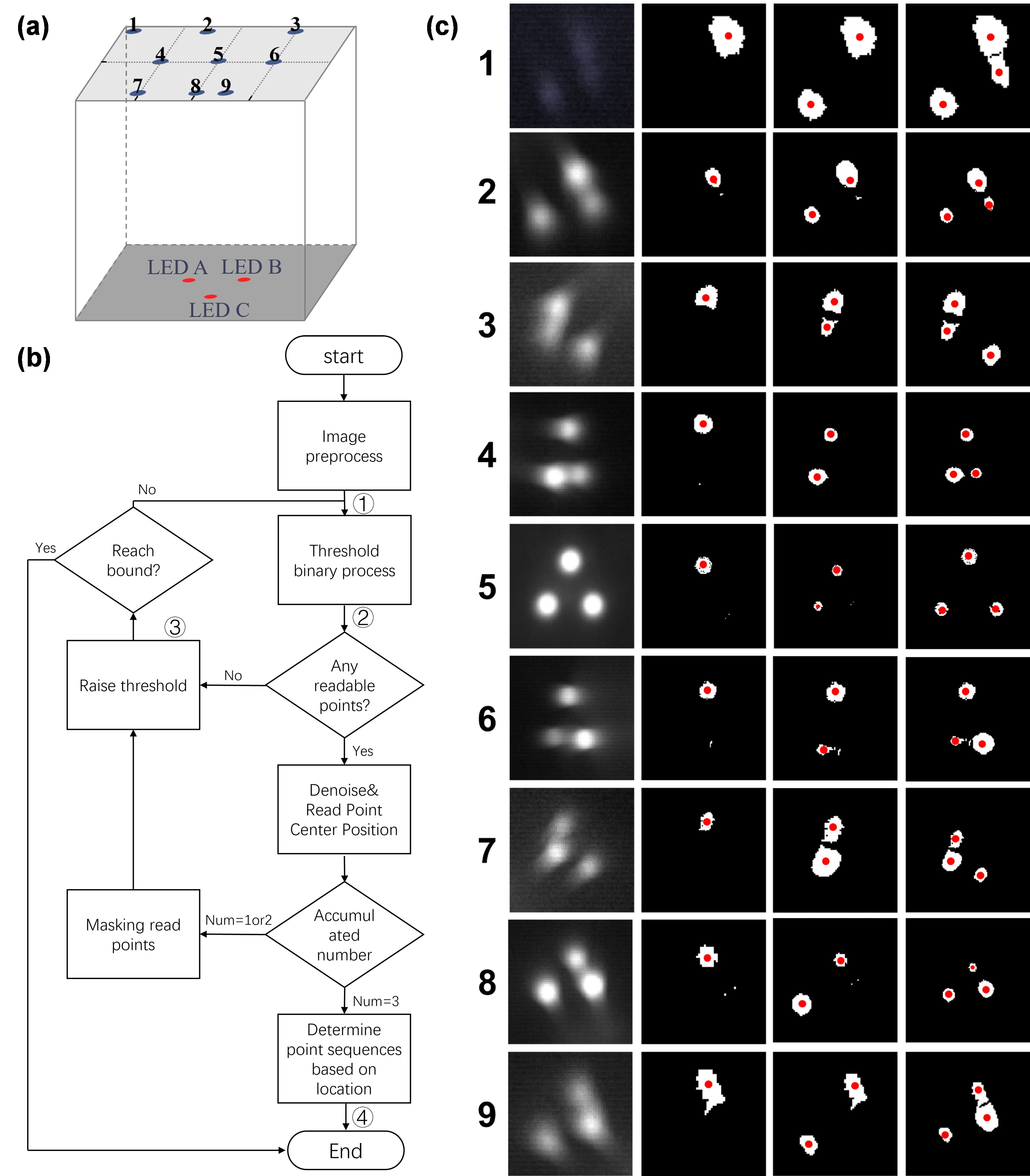


**Figure S4:** Detailed schematic diagram of point-by-point threshold algorithm in wide-angle imaging center extraction. (a) Schematic diagram of the location of point-by-point threshold algorithm show points in (c). (b) Flow chart of point-by-point threshold process. (c) The processed results generated by the point-by-point threshold algorithm at different angles of the model in (a), with each row corresponding to the position with the same row number in (a).

**Note S5 Positioning experiment on another height plane**

In order to obtain the influence of incident angle on positioning accuracy, we also conducted a positioning experiment at the MPD height of H = 1m, with a model size of 1m × 0.5m, which is the same as the size of the main text experiment zone. At a height of 1m, the maximum angle of the edge of the model can reach 30°. The average error is shown in the table below, and the distribution of average positioning accuracy is shown in Fig. S5.

**Table S2. Average positioning error of experiment height of 1m**

|  | X | Y | Z | Total |
| --- | --- | --- | --- | --- |
| average | 0.0018m | 0.0015m | 0.0104m | 0.0106m |
| 90%min | 0.0014m | 0.0012m | 0.0097m | 0.0098m |
| 80%min | 0.0012m | 0.0011m | 0.0093m | 0.0094m |
| 70%min | 0.0011m | 0.00097m | 0.0090m | 0.0091m |


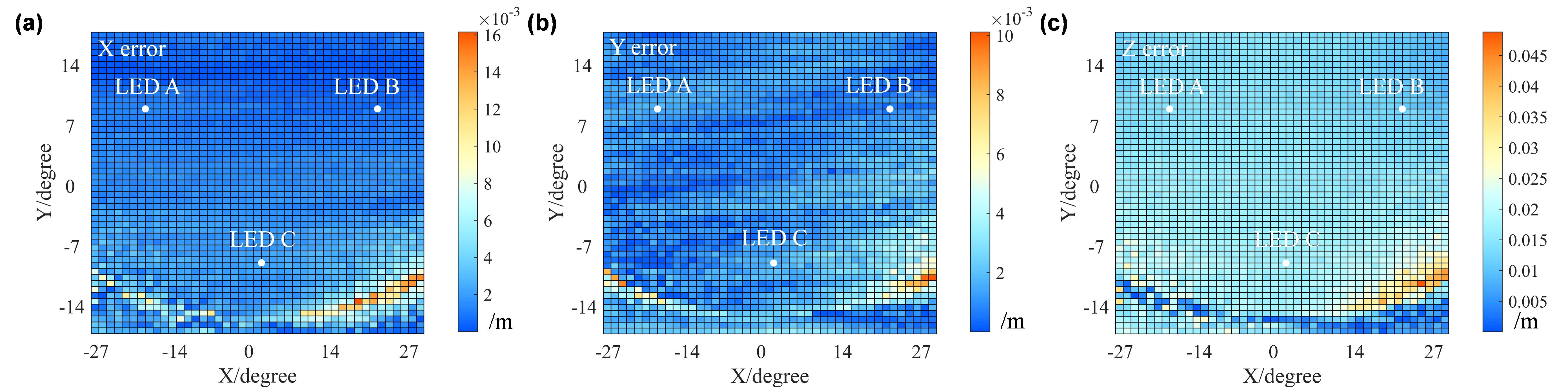


**Figure S5:** Schematic diagram of MPD positioning error distribution of experiment height of 1m. X-axis error is presented in (a), Y-axis in (b) and Z-axis in (c).

**Note S6 Positioning scheme that determines both attitude angles and three-dimensional positions**

According to Eq. (S3.1) in **Note S3**, it can be seen that without considering attitude rotation, the position of MPD satisfies the following expression

$$\begin{aligned} X_{i}=f\frac{\sqrt{\left( x-x_{i} \right)^{2}+\left( y-y_{i} \right)^{2}}}{\sqrt{\left( x-x_{i} \right)^{2}+\left( y-y_{i} \right)^{2}+\left( z-z_{i} \right)^{2}}}=f\frac{\left| OA \right|-\left( \left| OA \right|\cdot\vec{e_{z}} \right)\vec{e_{z}}}{\left| OA \right|}\#\left( S6.1 \right) \end{aligned}$$

where $\vec{e_{z}}$ is the normal vector of the x-o-y plane, $A(x_{i},y_{i},z_{i})$ is the LED position and $O(x,y,z)$ is the MPD position.

Figure S6(a) shows a schematic diagram of attitude rotation, where we define two attitude rotation angles, $\psi_{x}$ and $\psi_{y}$. $\psi_{x}$ is the positive pitch counter clockwise around x-axis, when looking towards the origin, down the x-axis and $\psi_{y}$ is the positive yaw counter clockwise around y-axis, when looking towards the origin, down the y-axis.

We assume that the metalens has an attitude angle, as shown in Fig. S6(b). This attitude can be obtained by rotating the initial attitude in the local coordinate system, and the attitude angle is synthesized by $\psi_{x}$ and $\psi_{y}$. The expression of the rotation matrix are as follows

$$\begin{aligned} R_{x}\left( \psi_{x} \right)=\left[ \begin{matrix} 1 & 0 & 0 \\ 0 & cos\psi_{x} & -sin\psi_{x} \\ 0 & sin\psi_{x} & cos\psi_{x} \end{matrix} \right]\#\left( S6.2 \right) \end{aligned}$$

$$\begin{aligned} R_{y}\left( \psi_{y} \right)=\left[ \begin{matrix} cos\psi_{y} & 0 & sin\psi_{y} \\ 0 & 1 & 0 \\ -sin\psi_{y} & 0 & cos\psi_{y} \end{matrix} \right]\#\left( S6.3 \right) \end{aligned}$$

$$\begin{aligned} R_{Total}= R_{y}\left( \psi_{y} \right)\cdot R_{x}\left( \psi_{x} \right)= \left[ \begin{matrix} cos\psi_{y} & sin\psi_{x}sin\psi_{y} & cos\psi_{x}sin\psi_{y} \\ 0 & cos\psi_{x} & sin\psi_{x} \\ -sin\psi_{y} & sin\psi_{x}cos\psi_{y} & cos\psi_{x}cos\psi_{y} \end{matrix} \right]\#\left( S6.4 \right) \end{aligned}$$

$R_{x}\left( \psi_{x} \right)$ and $R_{y}\left( \psi_{y} \right)$ represent the rotation matrices that rotate along the X-axis and Y-axis, respectively.

So we can apply the inverse of attitude rotation on point $A(x_{i},y_{i},z_{i})$ to obtain point $A^{'}(x_{i}',y_{i}',z_{i}')$ in local coordinate system.

$$\begin{aligned} A^{'}= {R_{Total}}^{-1}\cdot A= R_{y}\left( {-\psi}_{y} \right)\cdot R_{x}\left( {-\psi}_{x} \right)\cdot A\#\left( S6.5 \right) \end{aligned}$$

In global coordinate system

$$\begin{aligned} {OA}^{'}= {R_{Total}}^{-1}\cdot OA\#\left( S6.6 \right) \end{aligned}$$

For the light incident from point $A$ to the rotating metalens x' - y' - z', the incidence angle is the same as the light incident from point $A^{'}$ to the initial metalens x-y-z. Therefore, we can obtain the relationship that the MPD position satisfies under attitude rotation.

$$\begin{aligned} X_{i}=f\frac{\left| OA^{'} \right|-\left( \left| OA^{'} \right|\cdot\vec{e_{z}} \right)\vec{e_{z}}}{\left| OA^{'} \right|}=f\frac{\left| {R_{Total}}^{-1}\cdot OA \right|-\left( \left| {R_{Total}}^{-1}\cdot OA \right|\cdot\vec{e_{z}} \right)\vec{e_{z}}}{\left| {R_{Total}}^{-1}\cdot OA \right|}\#\left( S6.7 \right) \end{aligned}$$

It can be seen that there are five unknown variables in the relationship, so five equations are needed to eliminate degrees of freedom. A total of five LEDs are needed to determine the $(x,y,z,\psi_{x},\psi_{y})$ dimension of the MPD device.


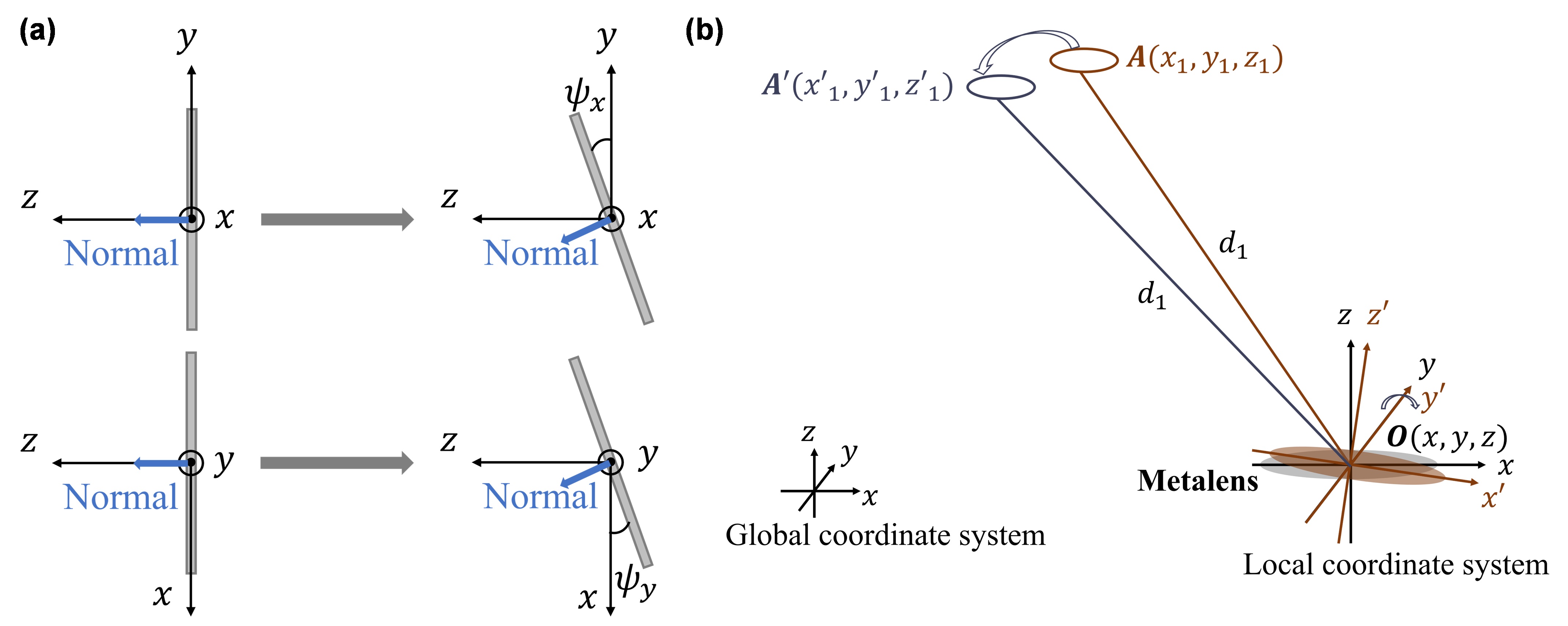


**Figure S6:** Schematic diagram of attitude rotation. (a) Schematic diagram of attitude rotation angle $\psi_{x}$ and $\psi_{y}$. (b) Schematic diagram of MPD rotation, and the rotation of $A$ to $A'$ in local coordinate system.

**Note S7 Extending the positioning scheme into large-scale indoor scenes.**

Positioning in a room of several meters in size is sufficient for indoor optical communication, but for navigation in scenes such as large shopping malls or underground parking lots, the positioning scheme is required to be effective at a size of hundreds of meters. Our positioning scheme is scalable in terms of the room size by increasing the numbers of LEDs on the ceiling, and can be expanded to meet the needs of large-size positioning. Figure S7 shows the top view of a large-scale indoor area, which is composed of unit cells with side length of 2.4m (Unit size is a proportional extension of the experimental model in the main text). Each LED has the identity (ID) information as shown in Fig. S7, which is used to indicate the coordinates of the LED at the cell. The ID information is coded in the flashing of the LED. By adjusting the CMOS shutter frequency comparable to the LED flashing frequency, stripes patterns will appear on the CMOS, through measuring the parameters of which, the ID information would be obtained (see Fig. S7). When a MPD is implemented for navigation in this room, multiple signal LEDs would be imaged. By measuring the one closest to the CMOS center, which unit cell area the MPD is in, would be known. Then using the coordinates of LEDs at this area the accurate position of the MPD would be calculated.


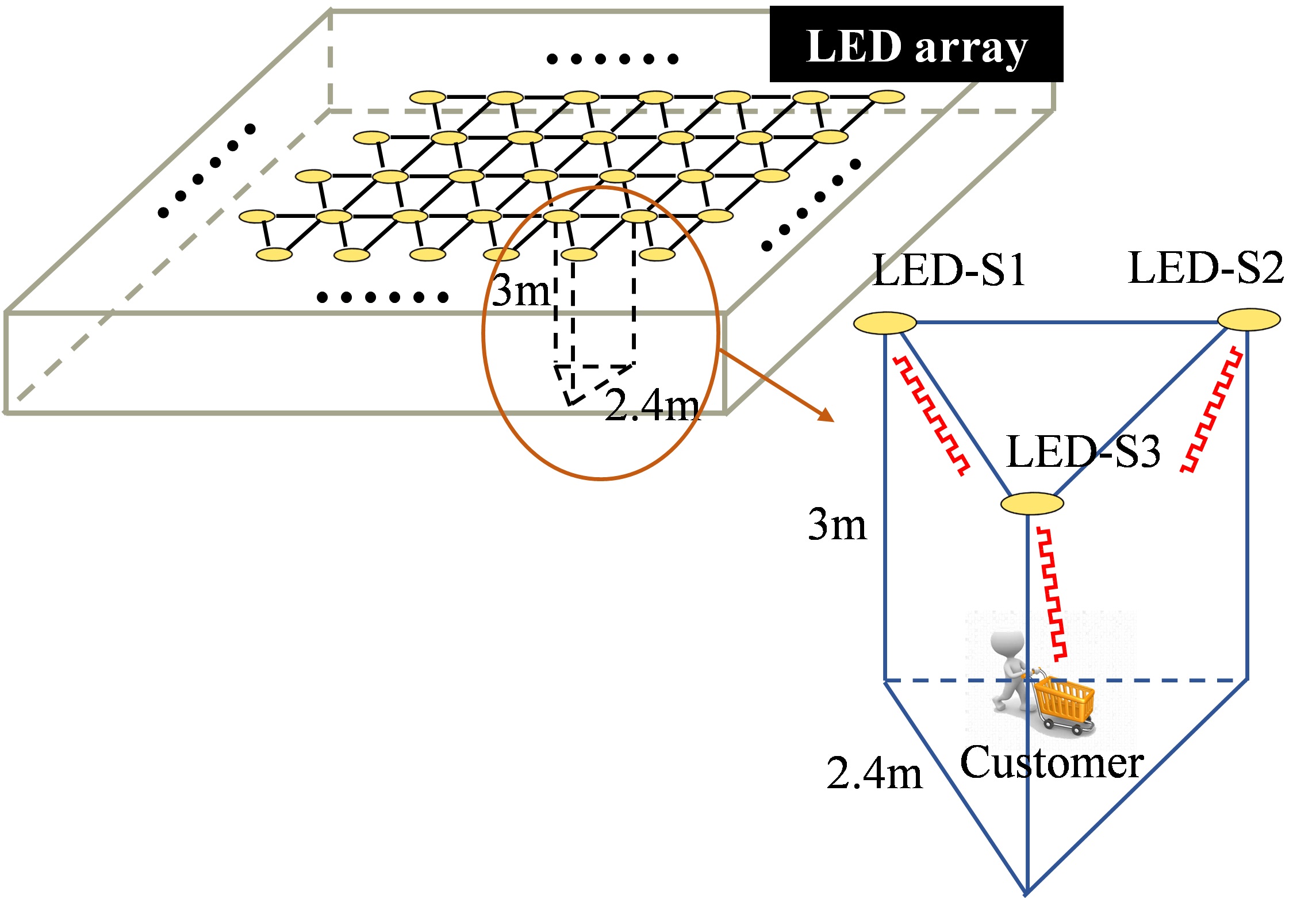


**Figure S7:** Schematic diagram of extending the positioning scheme into large-scale indoor scene.

**Note S8** **Comparison of focusing performances between metalens and traditional lens**

A lens is a critical component of the imaging-based positioning systems. For a commonly used traditional lens with a spherical phase, the phase expression is:

$$\begin{aligned} \phi_{sph}=\left\{ \begin{matrix} -4\pi n_{0}\Delta n\left( R-\sqrt{R^{2}-r^{2}} \right)/\lambda_{0}, r\leq R \\ 0 , r>R \end{matrix} \right.\#\left( S8.1 \right) \end{aligned}$$

where $\lambda_{0}$ is the free space wavelength, r is the distance to the lens’ center, R is the radius of curvature, $n_{0}$ is the refractive index of air, and Δn is the refractive index contrast between the lens and the air. The focal distance f of the spherical lens is given by the ratio of R and Δn:

$$\begin{aligned} f=\frac{R}{2\Delta n}.\#\left( S8.2 \right) \end{aligned}$$

Figure S8(a-c) show the Zemax OpticStudio ray tracing results of light incident on spherical phase traditional lens at 0°, 30°, and 60°, respectively. According to the simulation results, the convergence position of light rays and the shape of the focusing spot of a traditional spherical phase lens change significantly under oblique incidence.

To addressing the limitations of spherical phase lens under varying angles of incidence. The expression for the quadratic phase can be derived by taking the limits of the spherical phase parameters R and Δn,

$$\begin{aligned} \phi_{qua}=\lim_{\left( R,\Delta n \right)\to(+\infty,+\infty)} -\frac{4\pi}{\lambda_{0}}n_{0}\Delta n\left( R-\sqrt{R^{2}-r^{2}} \right)=-\frac{\pi r^{2}}{\lambda_{0}f}n_{0}.\#\left( S8.3 \right) \end{aligned}$$

Figure S8(d-f) demonstrates that the quadratic phase traditional lens has better convergence and imaging results compared to the spherical phase traditional lens. Theoretically, for oblique incidence at any angle, a quadratic phase traditional lens can produce the same focusing spot when the lens' outgoing light is in the same plane. However, due to the curved shape of traditional lenses, the focusing position of quadratic phase traditional lenses still change when the light obliquely incident at different angles, as shown in Figure S8(d-f), resulting in different shapes of focusing spots.

In contrast, for a flat quadratic phase metalens, the focusing position remains on the same plane under oblique incidence, as illustrated in Figure S8(g-i). Consequently, a quadratic phase metalens can achieve a consistent focusing spot shape at any angle of oblique incidence. It is important to note that a traditional bulk lens with a quadratic phase is not equivalent to a metalens, as their optical characteristics only coincide for paraxial rays.

Therefore, compared with traditional lens systems, the quadratic phase metalens system has a larger field of view (FOV) and superior performance for large-angle imaging. Since the positioning algorithm relies on the images formed by the lens system, the quadratic phase metalens system can achieve higher positioning accuracy than traditional lens systems within a large FOV.


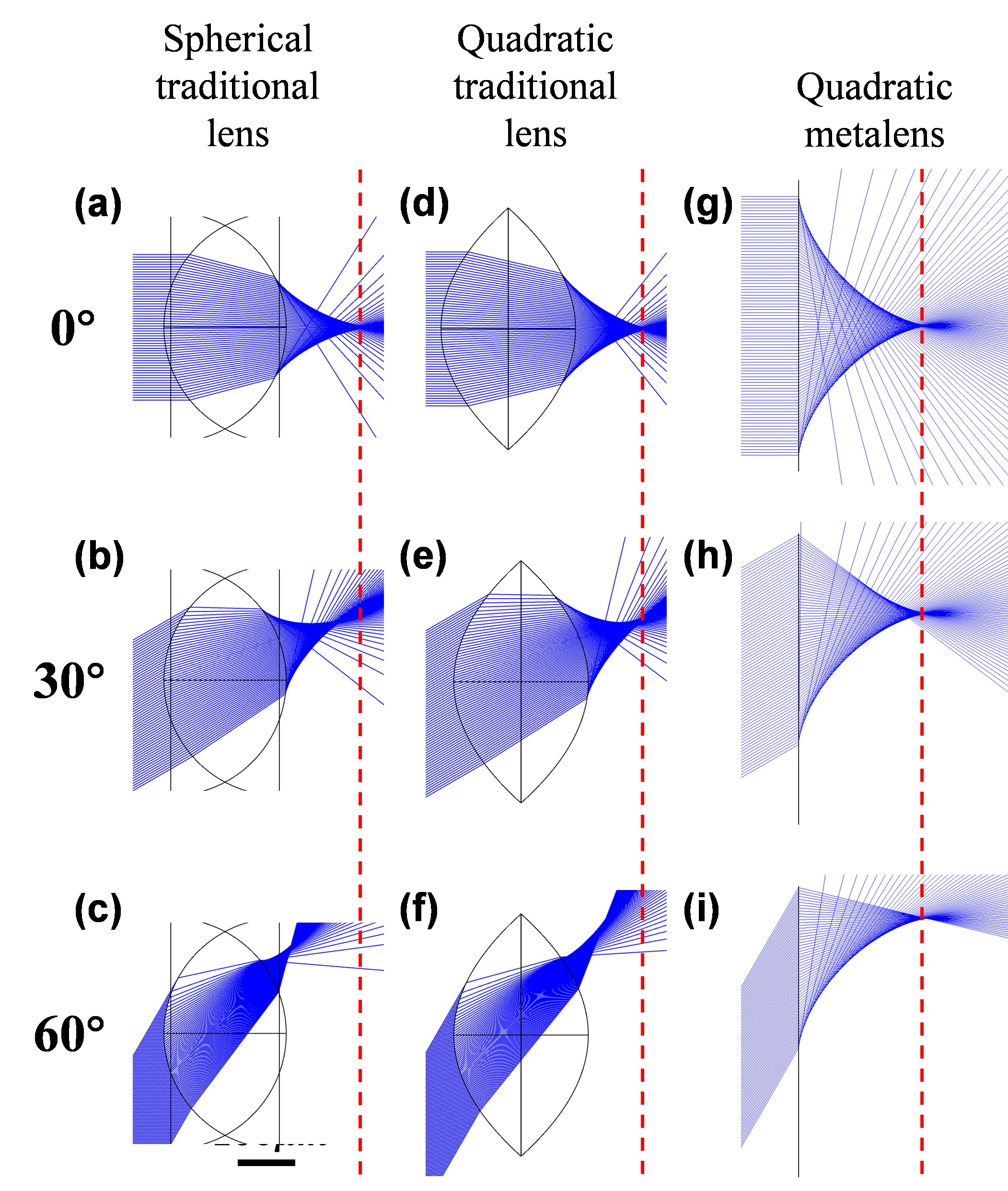


**Figure S8:** Zemax OpticStudio ray tracing simulation of traditional lens and metalens. Focusing performances of (a-c) spherical phase traditional lens at 0°, 30°, 60° incidence, (d-f) quadratic phase traditional lens at 0°, 30°, 60° incidence, and (g-i) quadratic phase metalens at 0°, 30°, 60° incidence. The red dashed lines mark lenses’ the focal position at normal incidence.
